# Clustering of lifestyle behaviours and analysis of their associations with MAFLD: a cross-sectional study of 196,515 individuals in China

**DOI:** 10.1186/s12889-023-17177-3

**Published:** 2023-11-21

**Authors:** Bingqian Zhou, Ni Gong, Qingnan He, Xinjuan Huang, Jingchi Zhu, Lijun Zhang, Yanyan Huang, Xinyun Tan, Yuanqin Xia, Yu Zheng, Qiuling Shi, Chunxiang Qin

**Affiliations:** 1 Department of Health Management Center, The Third Xiangya Hospital, Central South University, No.138, Tongzipo Road, Changsha, 410013 China; 2 Xiangya Nursing School, Central South University, Changsha, 410013 China; 3 Jishou University School of Medicine, Jishou, 416000 China; 4 State Key Laboratory of Ultrasound in Medicine and Engineering, School of Public Health and Management, Chongqing Medical University, No.1, Medical College Road , Chongqing, 400016 China

**Keywords:** Cluster analysis, Multivariate analysis of variance, Principal component analysis, Lifestyle scoring system, Lifestyle pattern, MAFLD

## Abstract

**Background:**

The aggregation of lifestyle behaviours and their association with metabolic-associated fatty liver disease (MAFLD) remain unclear. We identified lifestyle patterns and investigated their association with the risk of developing MAFLD in a sample of Chinese adults who underwent annual physical examinations.

**Methods:**

Annual physical examination data of Chinese adults from January 2016 to December 2020 were used in this study. We created a scoring system for lifestyle items combining a statistical method (multivariate analysis of variance) and clinical expertise (Delphi method). Subsequently, principal component analysis and two-step cluster analysis were implemented to derive the lifestyle patterns of men and women. Binary logistic regression analysis was used to explore the prevalence risk of MAFLD among lifestyle patterns stratified by sex.

**Results:**

A total of 196,515 subjects were included in the analysis. Based on the defined lifestyle scoring system, nine and four lifestyle patterns were identified for men and women, respectively, which included “healthy or unhealthy” patterns and mixed patterns containing a combination of healthy and risky lifestyle behaviours. This study showed that subjects with an unhealthy or mixed pattern had a significantly higher risk of developing MAFLD than subjects with a relatively healthy pattern, especially among men.

**Conclusions:**

Clusters of unfavourable behaviours are more prominent in men than in women. Lifestyle patterns, as important factors influencing the development of MAFLD, show significant sex differences in the risk of MAFLD. There is a strong need for future research to develop targeted MAFLD interventions based on the identified behavioural clusters by sex stratification.

**Supplementary Information:**

The online version contains supplementary material available at 10.1186/s12889-023-17177-3.

## Introduction

Metabolic-associated fatty liver disease (MAFLD), formerly named nonalcoholic fatty liver disease (NAFLD), is a multisystemic metabolic disorder involving the liver [1]. MAFLD affects more than 25% of the world’s adult population and 29%–46% of the Chinese population, and the prevalence is increasing dramatically every year, with a significant impact on health and economic burden for all of society [1–3]. Tremendous plasticity is observed in MAFLD progression over the lifespan. While nonmodifiable mechanisms (i.e., genetic predisposition) are partly to blame, it is plausible that modifiable lifestyles have a substantial impact on the development of MAFLD [4].

The rapid rise in sedentary behaviour, physical inactivity, and excess energy intake relative to expenditure due to nutritional imbalance and unhealthy dietary behaviours have individually been associated with MAFLD [5, 6]. However, their effects on individuals’ health and disease were multifactorial and interrelated in synergistic or even cumulative manners. Investigating the comprehensive impact of these lifestyle behaviours as a whole, rather than focusing on a single behaviour or dimension, is necessary [7]. The “lifestyle pattern,” as an alternative approach to only focusing on the health effects of certain behaviours or lifestyles in nutritional epidemiology research, has been adopted as an influential factor in previous studies on adiposity [8], metabolic syndrome [9], and hypertension [10]. However, the lifestyle variables in these studies focused on certain lifestyle behaviours (e.g., diet, physical activity, and sedentary behaviours) and less on sleep or mental health dimensions, which were shown to be independently related to MAFLD [11, 12]. Few studies have reported the association between lifestyle patterns and MAFLD. Investigating the holistic “lifestyle patterns” that encompass a broad spectrum of lifestyle behaviours and analysing their contribution to MAFLD is necessary to capture “real-life” associations and implications.

The Health Checkup Self-Assessment Questionnaire (HCSAQ), a widely used tool for physical examination data collection in the field of health management in China, incorporates a more comprehensive list of lifestyle behaviours than most existing lifestyle evaluation instruments [13]. Although five subscale scores of this questionnaire were developed and validated [14], no standardized lifestyle scoring rules for overall lifestyle items are available, limiting the questionnaire’s application in identifying the populations at risk for behaviour-related diseases.

Therefore, we analysed the HCSAQ data from a large cohort of physical examinations to 1) generate a lifestyle scoring system to identify and characterize lifestyle patterns of men and women separately in a sample of the Chinese adult physical examination population (inspired by sex-specific differences in lifestyle behaviours and MAFLD prevalence) and 2) examine the association between these sex-stratified lifestyle patterns and the risk of developing MAFLD.

## Methods

### Study design and participants

**Fig. 1:**
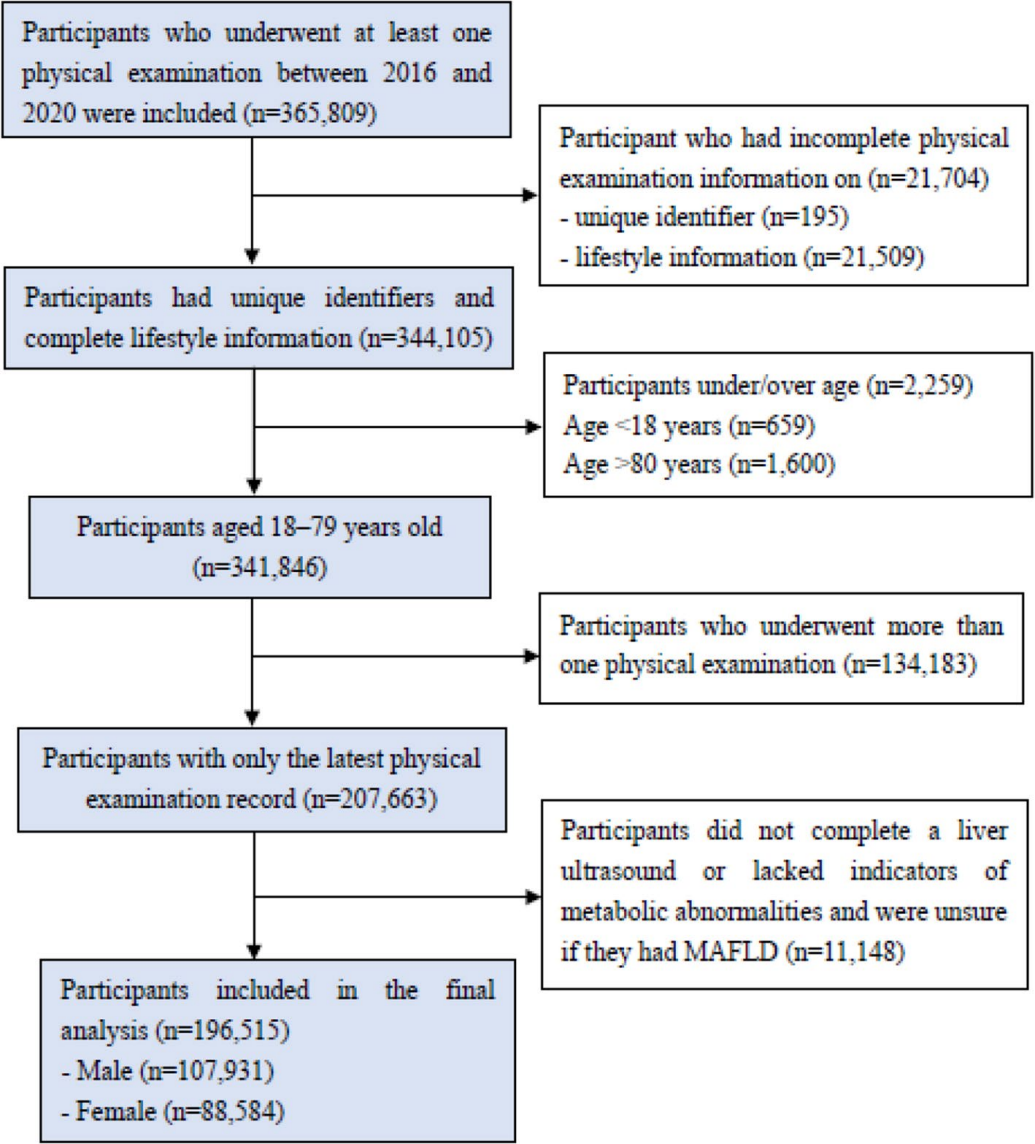
The flow chart of the participant selection procedure

A cross-sectional analysis was performed on data obtained from the Health Management Center of a large general hospital between January 2016 and December 2020 in Hunan, China. Physical examination data were collected from participants who underwent annual physical examinations, including sociodemographics, self-reported lifestyle behaviours, and clinical and laboratory data. Potential bias was controlled by including all physical examination data recorded for that period rather than conducting random sampling, except for the following situations: i) participants lacking self-reported lifestyle behaviours and unique identifiers, ii) participants younger than 18 years or older than 79 years (first, the HCSAQ is for adults 18 years of age or older; second, the sample of older individuals has poor or no self-care ability, and their lifestyle information is incomplete or inaccurate between the ages of 80 and 99 years), and iii) participants without reliable MAFLD diagnostic results. For participants undergoing more than one examination during the study period, only the latest examination was included. The selection procedure for the study subjects is shown in Fig. 1. Our final study population consisted of 196,515 individuals aged 18 to 79 years living in China.

### Sociodemographic assessment, clinical and laboratory data measurement

Sociodemographic information of the study population included age, sex, educational level, marital status, occupation category, and type of medical insurance.

Clinical and laboratory data were collected by experienced clinicians and trained ultrasonographers using standard techniques, including the following: (i) anthropometric measurements (including blood pressure, weight, height, and waist circumference [WC]) and body mass index (BMI), calculated as weight (kg) divided by the square of height (m) (kg/m^2^); (ii) laboratory tests (including fasting blood glucose, fasting insulin, HbA1c, 2-h postload glucose, plasma triglycerides (TG), plasma HDL-cholesterol (HDL-C), and plasma high-sensitivity C-reactive protein (hs-CRP)); and (iii) liver and abdominal ultrasound.

### Self-reported lifestyle behaviours

The relevant lifestyle behaviour items were derived from the HCSAQ, which was published in Expert Consensus on Basic Items of Health Checkup in 2014 [13]. The questionnaire contains six dimensions: health history, somatic symptoms, lifestyle habits, mental stress, sleep quality, and health literacy, with 87 specific items to collect the comprehensive personal health and disease information of the examinee. The questionnaire has been promoted and applied in multiple authoritative physical examination centres in China. The present study defined a healthy lifestyle as the organic unity of physical and mental behaviours [15]. Therefore, we focus on lifestyle habits (including nutrition, physical exercise, smoking, and drinking), mental stress, and the sleep quality of the HCSAQ. Detailed information regarding lifestyle behaviours is presented in Appendix Table S1 in the Supplementary Materials.

### Assessment of MAFLD

According to the latest criteria [16], the diagnosis of MAFLD in this study is based on ultrasonically confirmed hepatic steatosis and one of the following three criteria: overweight or obesity (defined as BMI > 23 kg/m^2^ in Asians), presence of type 2 diabetes mellitus (T2DM), or evidence of metabolic dysregulation. Metabolic dysregulation was defined as the presence of ≥ 2 of the following criteria [1]: (i) WC ≥ 90/80 cm (Asian cut-off) in men/women; (ii) blood pressure ≥ 130/85 mmHg or specific drug treatment; (iii) TG ≥ 1.7 mmol/L or specific drug treatment; (iv) HDL-C < 1.0 mmol/L in men and < 1.3 mmol/L in women; (v) prediabetes (i.e., FPG of 5.6 to 6.9 mmol/L or HbA1c of 5.7% to 6.4% or 2-h postload glucose level of 7.8 to 11.0 mmol); (vi) HOMA-IR score ≥ 2.5; and (vii) Hs-CRP level > 2 mg/L. The diagnosis of hepatic steatosis on ultrasound was based on the presence of hepatorenal echo contrast, liver parenchymal brightness, deep attenuation, and vascular blurring [17].

### Statistical analysis

**Fig. 2:**
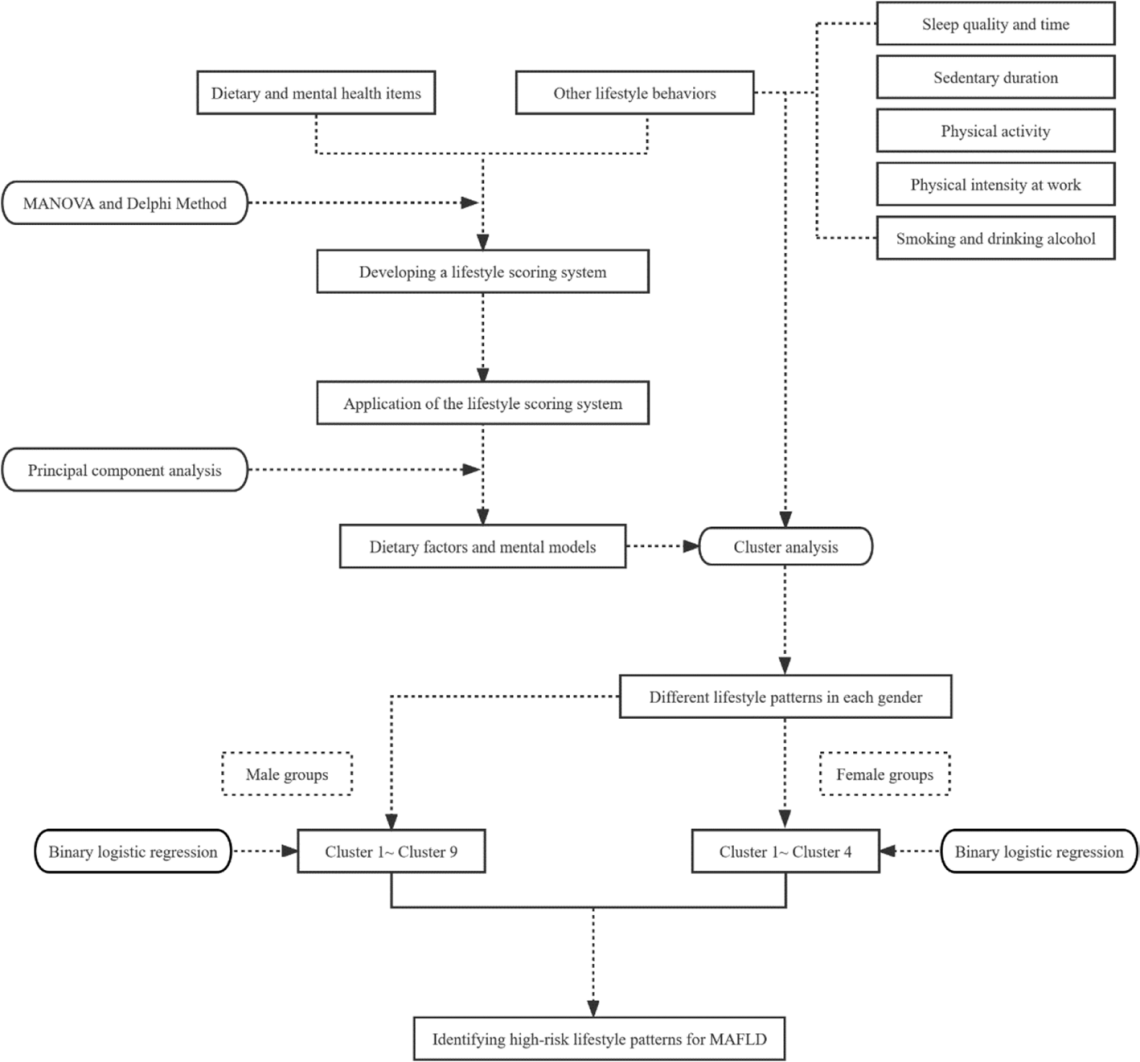
The flow chart of statistical analysis

The flow chart of the statistical analysis is shown in Fig. 2. The sociodemographics of the sample are described as frequencies (percentages).

### Empirical derivation of lifestyle patterns

#### Establishing a lifestyle scoring system

Given the lack of lifestyle scoring rules in the HCSAQ, a standardized normative scoring system for lifestyle items was established using multivariate analysis of variance (MANOVA) and the Delphi method.

MANOVA was conducted to derive the statistically optimal cut points for each lifestyle item according to the cut point analysis method of Wang et al. [18]. A total of eight interference variables (BMI, systolic blood pressure, diastolic blood pressure, TG, HDL-C, LDL-C, total cholesterol, and fasting glucose) were used as dependent (continuous) variables, and each lifestyle item was used as an independent (categorical) variable based on different potential cut points. The F ratio of MANOVA was used as a function that adequately reflects the relationship between lifestyle scores and health status. The optimal cut-off point of each lifestyle item is based on the largest F ratio by the criteria of Serlin et al. [19]. Based on the derived cut-off points, a numeric scale of − 3 to 3 for options of each lifestyle item was defined in conjunction with the healthy lifestyle guidelines [15].

Next, two rounds of Delphi expert consultation were conducted, and experts in epidemiology and health statistics, health management, nutrition, kinesiology, and psychology were invited to select optimal boundaries for categorizing options for each lifestyle item. Ultimately, the cut points of options for lifestyle items that satisfied the top three F-ratios in MANOVA and the top three choice times by experts were considered the most appropriate to constitute the lifestyle scoring system. The defined scoring system was applied to convert lifestyle information into reasonable ordinal variables. The higher the level of engagement in healthy lifestyle behaviours, the higher the score. For unhealthy lifestyles, the score is the opposite (more points are given for lower engagement).

#### Identifying different lifestyle patterns

Principal component analysis (PCA) was performed to reduce the dimensionality of diet and mental health. Dietary factors and mental models were extracted based on scree plots and professional interpretability [20]. Subsequently, smoking and alcohol consumption status, physical activity, physical intensity at work, sedentary lifestyle, sleep, mental models, and dietary factors were included in the cluster analysis (CA) to identify distinct lifestyle patterns. Variables were transformed into standardized scores (Z scores) before data analysis to provide a common range because of different units and arithmetic scales [21].

CA is divided into two steps [22]. First, hierarchical cluster analysis was carried out using Ward’s method based on squared Euclidean distances [23]. Second, an iterative on-hierarchical K-means clustering procedure was used to identify groups of subjects with common lifestyle behaviours. Initial cluster centres based on Ward’s hierarchical method were used as nonrandom starting points. The optimal clustering solution fulfils the intercluster maximum dissimilarity and the interpretability of clusters. Finally, the silhouette coefficient and elbow method were used to assess the quality of the clustered solutions. To assess the stability and reliability of the clustering solutions, an internal random subsample (50% of the total study sample) of each sex was clustered by performing the same procedure (Ward and K-means steps). The Cohen k coefficient was used to assess the reliability of the cluster solutions (> 0.75 indicates good consistency) [24]. Clusters were computed specifically for males and females because of sex differences in lifestyle behaviours and the risk of MAFLD. The differential features of each cluster were identified by comparing the mean Z scores at the centre of the cluster, and Z scores were positively correlated with the healthiness of lifestyle behaviours. Statistical differences among the clusters were analyzed by Chi-square (categorical variables).

The associations between the clusters with distinct lifestyle patterns (independent variables) and MAFLD (dependent variables) were investigated in the study population using binary logistic regression models with adjustment for relevant covariates, including age, education level, marital status, occupational category, and type of health insurance. Outliers and missing values were corrected and added by rechecking the original data in the data management system. The variables with severe missingness were included as extra categories in the corresponding covariates by referring to the statistical analysis method in the study of Jiayao Lei et al. when they analysed HPV vaccination and the risk of invasive cervical cancer [25]. A *P* value < 0.05 (two-sided) was considered indicative of statistical significance. All analyses were performed using the statistical software SAS (version 9.4).

## Results

The final analysis included 196,515 subjects. The sociodemographic and lifestyle characteristics of the male and female samples are presented separately in Table 1. Males accounted for 54.92% of the sample, whereas females comprised 45.08%. The proportion of middle-aged and elderly people aged ≥ 45 years is 45.55%, and the proportion of young people aged < 45 years old is 54.45%. A total of 77,172 cases of MAFLD (39.27%) were identified between 2016 and 2020, and men had a prevalence of 54.37%, which was higher than that of women (20.88%, *p* < 0.001).

**Table 1:** Descriptive statistics on the sociodemographic and lifestyle characteristics of participants separately by sex

Sociodemographic (N, %)	Total (*N* = 196,515)	MAFLD (*N* = 77,172)	Non-MAFLD (*N* = 119,343)	*χ*^*2*^	*P*
**Age**				6369.318	< 0.001
< 45	107,008(54.45)	33,418(43.30)	73,590(61.66)		
≥ 45	89,507(45.55)	43,754(56.70)	45,753(38.34)		
**Sex**					< 0.001
Male	107,931(54.92)	58,678(76.04)	49,253(41.27)	22,879.215	
Female	88,584(45.08)	18,494(23.96)	70,090(58.73)		
**Education level**					< 0.001
Junior high school and below	20,169(10.26)	8404(10.89)	11,765(9.86)		
Senior high school	27,098(13.79)	11,628(15.07)	15,470(12.96)	279.473	
College and above	99,259(50.51)	37,608(48.73)	61,651(51.66)		
Missing data	49,989(25.44)	19,532(25.31)	30,457(25.52)		
**Marital status**					< 0.001
Married	168,076(85.53)	70,400(91.22)	97,676(81.84)		
Unmarried	21,668(11.03)	4492(5.82)	17,176(14.39)	3814.046	
Divorced/widowed	4740(2.41)	1798(2.33)	2942(2.47)		
Missing data	2031(1.03)	482(0.62)	1549(1.30)		
**Occupation category**					< 0.001
Mental labour	72,295(36.79)	28,510(36.94)	43,785(36.69)		
Physical labour	42,946(21.85)	17,034(22.07)	25,912(21.71)	41.626	
Other	31,597(16.08)	11,902(15.42)	19,695(16.50)		
Missing data	49,677(25.28)	19,726(25.56)	29,951(25.10)		
**Type of medical insurance**					< 0.001
Urban resident medical insurance	13,473(6.86)	5240(6.79)	8233(6.90)		
Urban worker medical insurance	105,335(53.60)	42,081(54.53)	63,254(53.00)	156.622	
Agricultural cooperative	19,689(10.02)	7552(9.79)	12,137(10.17)		
No medical insurance	3167(1.61)	935(1.21)	2232(1.87)		
Missing data	54,851(27.91)	21,364(27.68)	33,487(28.06)		

The defined lifestyle scoring system, including optimal cut points for the options of the lifestyle items, is presented in Appendix Table S1 in the Supplementary materials. The factors extracted by the PCA method corresponded to dietary factors and mental models. The factor loadings for each dietary factor are shown in Appendix Table S2 in the Supplementary materials.

**Fig. 3:**
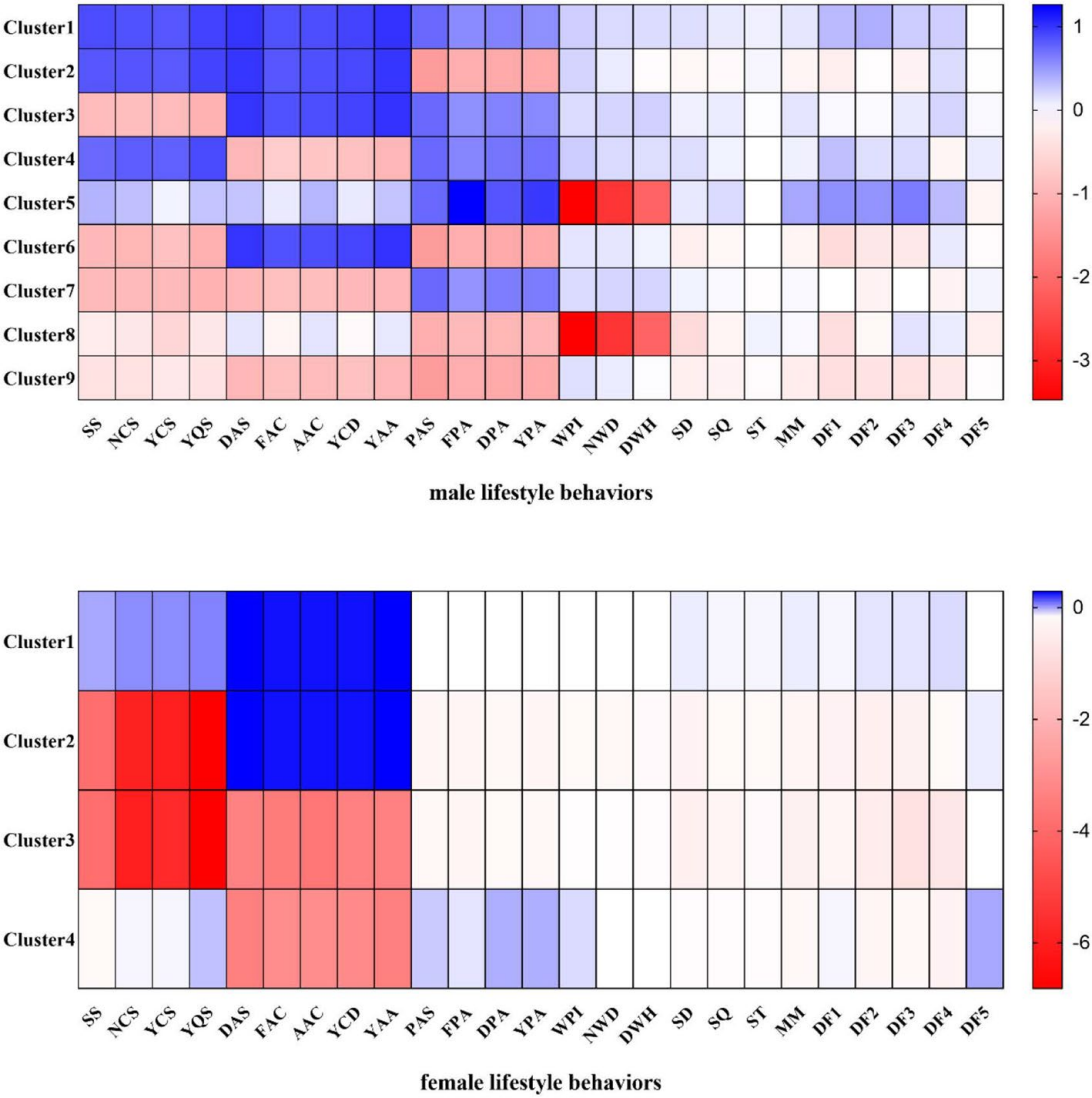
Lifestyle clusters of males and females (Z scores). Note: YPA: Years of consistent exercise, SS: Smoking status, WPI: Physical intensity at work, NCS: Number of cigarettes smoked per day, NWD: Number of working days per week, YCS: Number of years of continuous smoking, DWH: Daily working hours, YQS: Number of years to quit smoking, SD: Sedentary duration, DAS: Drinking alcohol status, SQ: Sleep quality, FAC: Frequency of alcohol consumption per week, ST: Sleep time, AAC: Amount of alcohol consumption per drink, MM: Mental model, YCD: Number of years of continuous drinking, DF1: Dietary Factor 1, YAA: Number of years of alcohol abstinence, DF2: Dietary Factor 2, PAS: Physical activity status, DF3: Dietary Factor 3, FPA: Frequency of exercise per week, DF4: Dietary Factor 4, DPA: Duration of each exercise, DF5: Dietary Factor 5

Based on the results of the silhouette coefficient and elbow method, we identified nine clusters for males and four clusters for females. A relatively high agreement was found between the cluster solution derived from the full sample and the random subsample (males: Cohen’s kappa = 0.55, *p* < 0.01; females: Cohen’s kappa = 0.89, *p* < 0.01). The specific characteristics of each cluster are presented in Fig. 3 and Table 2.

**Table 2:** Sociodemographic characteristics of lifestyle clusters

**Males (n, %)**	Cluster 1 (*n* = 21,494, 19.91%)	Cluster 2 (*n* = 10,790, 10.00%)	Cluster 3 (*n* = 10,092, 9.35%)	Cluster 4 (*n* = 16,160, 14.97%)	Cluster 5 (*n* = 3917, 3.63%)	Cluster 6 (*n* = 7656, 7.09%)	Cluster 7 (*n* = 17,723, 16.42%)	Cluster 8 (*n* = 1865, 1.73%)	Cluster 9 (*n* = 18,234, 16.89%)	***χ***^***2***^
**Age**										
< 45	11,889(55.31)	7844(72.70)	4711(46.68)	8040(49.75)	195(4.98)	4538(59.27)	8485(47.88)	337(18.07)	11,400(62.52)	7469.931***
≥ 45	9605(44.69)	2946(27.30)	5381(53.32)	8120(50.25)	3722(95.02)	3118(40.73)	9238(52.12)	1528(81.93)	6834(37.48)	
**Education level**										
Junior school and below	942(4.38)	869(8.05)	757(7.50)	684(4.23)	646(16.49)	1009(13.18)	1187(6.70)	550(29.49)	2064(11.32)	5388.775***
Senior high school	1974(9.18)	1108(10.27)	1573(15.59)	1491(9.23)	970(24.76)	1356(17.71)	2647(14.94)	554(29.71)	2935(16.10)	
College and above	12,673(58.96)	5809(53.84)	5462(54.12)	9979(61.75)	1634(41.72)	3534(46.16)	10,060(56.76)	450(24.13)	9254(50.75)	
Missing data	5905(27.47)	3004(27.84)	2300(22.79)	4006(24.79)	667(17.03)	1757(22.95)	3829(21.60)	311(16.68)	3981(21.83)	
**Marital status**										
Married	18,017(83.82)	8874(82.24)	8924(88.43)	14,620(90.47)	3692(94.26)	6697(87.47)	15,907(89.75)	1661(89.06)	16,184(88.76)	1542.946***
Unmarried	3085(14.35)	1725(15.99)	921(9.13)	1290(7.98)	116(2.96)	821(10.72)	1439(8.12)	120(6.43)	1685(9.24)	
Divorced/widowed	241(1.12)	87(0.81)	208(2.06)	183(1.13)	98(2.50)	106(1.38)	312(1.76)	80(4.29)	303(1.66)	
Missing data	151(0.70)	104(0.96)	39(0.39)	67(0.41)	11(0.28)	32(0.42)	65(0.37)	4(0.21)	62(0.34)	
**Occupation category**										
Mental labour	8349(38.84)	3634(33.68)	4327(42.88)	7479(46.28)	856(21.85)	2814(36.76)	8251(46.56)	321(17.21)	7296(40.01)	8779.203***
Physical labour	5049(23.49)	3213(29.78)	2294(22.73)	3208(19.85)	408(10.42)	2169(28.33)	3800(21.44)	382(20.48)	4791(26.28)	
Other	2161(10.05)	979(9.07)	1144(11.34)	1423(8.81)	1967(50.22)	890(11.62)	1719(9.70)	831(44.56)	2084(11.43)	
Missing data	5935(27.61)	2964(27.47)	2327(23.06)	4050(25.06)	686(17.51)	1783(23.29)	3953(22.30)	331(17.75)	4063(22.28)	
**Type of medical insurance**										
Urban resident medical insurance	1150(5.35)	650(6.02)	585(5.80)	894(5.53)	299(7.63)	564(7.37)	1139(6.43)	220(11.80)	1262(6.92)	2865.047***
Urban work medical insurance	12,480(58.06)	5519(51.15)	6034(59.79)	9809(60.70)	2556(65.25)	3779(49.36)	10,640(60.03)	840(45.04)	9554(52.40)	
Agricultural cooperative medical insurance	1119(5.21)	1164(10.79)	762(7.55)	808(5.00)	229(5.85)	1157(15.11)	1350(7.62)	363(19.46)	2528(13.86)	
No medical insurance	256(1.19)	202(1.87)	135(1.34)	142(0.88)	40(1.02)	167(2.18)	212(1.20)	59(3.16)	345(1.89)	
Missing data	6489(30.19)	3255(30.17)	2576(25.53)	4507(27.89)	793(20.25)	1989(25.98)	4382(24.72)	383(20.54)	4545(24.93)	
**MAFLD**										
Yes	10,332(48.07)	5385(49.91)	5433(53.83)	9431(58.36)	1998(51.01)	4147(54.17)	10,391(58.63)	975(52.28)	10,586(58.06)	786.207***
No	11,162(51.93)	5405(50.09)	4659(46.17)	6729(41.64)	1919(48.99)	3509(45.83)	7332(41.37)	890(47.72)	7648(41.94)	
**Females (n, %)**	Cluster 1 (*n* = 80,551, 90.93%)		Cluster 2 (*n* = 1040, 1.17%)		Cluster 3 (*n* = 718, 0.81%)		Cluster 4 (*n* = 6275, 7.08%)		***χ***^***2***^	
**Age**										
< 45	44,818(55.64)		582(55.96)		501(69.78)		3668(58.45)		74.821***	
≥ 45	35,733(44.36)		458(44.04)		217(30.22)		2607(41.55)			
**Education level**										
Junior school and below	10,371(12.88)		184(17.69)		102(14.21)		804(12.81)		137.908***	
Senior high school	11,153(13.85)		188(18.08)		153(21.31)		996(15.87)			
College and above	36,673(45.53)		446(42.88)		315(43.87)		2970(47.33)			
Missing data	22,354(27.75)		222(21.35)		148(20.61)		1505(23.98)			
**Marital status**										
Married	67,289(83.54)		760(73.08)		500(69.64)		4951(78.90)		452.223***	
Unmarried	9240(11.47)		164(15.77)		149(20.75)		913(14.55)			
Divorced/widowed	2612(3.24)		107(10.29)		65(9.05)		338(5.39)			
Missing data	1410(1.75)		9(0.87)		4(0.56)		73(1.16)			
**Occupation category**										
Mental labour	26,065(32.36)		334(32.12)		239(33.29)		2330(37.13)		140.534***	
Physical labour	16,184(20.09)		195(18.75)		120(16.71)		1133(18.06)			
Other	16,586(20.59)		287(27.60)		202(28.13)		1324(21.10)			
Missing data	21,716(26.96)		224(21.54)		157(21.87)		1488(23.71)			
**Type of medical insurance**										
Urban resident medical insurance	5977(7.42)		127(12.21)		80(11.14)		526(8.38)		178.411***	
Urban work medical insurance	40,184(49.89)		486(46.73)		326(45.40)		3128(49.85)			
Agricultural cooperative medical insurance	9220(11.45)		124(11.92)		97(13.51)		768(12.24)			
No medical insurance	1371(1.70)		44(4.23)		35(4.87)		159(2.53)			
Missing data	23,799(29.55)		259(24.90)		180(25.07)		1694(27.00)			
**MAFLD**										
Yes	16,760(20.81)		280(26.92)		151(21.03)		1303(20.76)		23.314***	
No	63,791(79.19)		760(73.08)		567(78.97)		4972(79.24)			

^***^ *P* < 0.001

For males, the largest cluster (C1, *n* = 21,494, 19.91%) was referred to as a “healthy” lifestyle pattern. It excluded all risky health behaviours and was characterized by being physically active, not smoking, not drinking, having low levels of sedentariness, having moderate physical activity at work, sleeping well, and having a healthy mind and diet. Cluster 2 (C2, *n* = 10,790, 10.00%) was characterized by physical inactivity, an unhealthy diet, not smoking, not drinking, being sedentary, and having poor mental status. Cluster 3 (C3, *n* = 10,092, 9.35%) was characterized by “heavy smoking” but maintaining other healthy lifestyle behaviours. Cluster 4 (C4, *n* = 16,160, 14.97%) was significantly characterized by heavily consuming alcohol, smoking in small quantities, being physically active, and having unhealthy dietary behaviours. Cluster 5 (C5, *n* = 3917, 3.63%) was described as having the “most physically intense work,” being physically active, having a healthy mentality, and having a high-fat and high-cholesterol diet. Cluster 6 (C6, *n* = 7656, 7.09%) was almost the exact opposite of Cluster 4 and was characterized by “heavy smoking,” being physically inactive, having “poor sleep quality,” and having an unhealthy diet but no alcohol consumption. Cluster 7 (C7, *n* = 17,723,16.42%) was the opposite of Cluster 2 and was characterized by “heavy smoking” and “heavy drinking” while also being physically active. Cluster 8 (C8, *n* = 1865,1.73%), similar to Cluster 5, was characterized by performing the “most physically intense work,” “heavy drinking,” “heavy smoking,” being physically inactive, being highly sedentary, and having an “unhealthy mentality and diet.” Last, Cluster 9 (C9, *n* = 18,234,16.89%), known as the “unhealthiest” lifestyle pattern, was characterized by heavy drinking and smoking, being physically inactive, being sedentary, having poor sleep quality, and having an unhealthy mental status and diet.

The largest cluster of females (C_F_1, *n *= 80,551, 90.93%), known as the “relatively healthy” lifestyle pattern, differed from the largest cluster of males in that they lacked physical activity. Cluster 2 (C_F_2, *n *= 1040, 1.17%) was characterized by heavy smoking, being highly sedentary, having poor mental status, and having an unhealthy diet. Cluster 3 (C_F_3, *n* = 718,0.81%), known as the “least healthy” lifestyle pattern, had similar characteristics to Cluster 2 except for heavy drinking. Cluster 4 (C_F_4, *n* = 6275, 7.08%) was characterized by heavy alcohol consumption but was the most active in exercise and had relatively healthy dietary behaviours.

In men, C4, C7, and C9 had the highest prevalence of MAFLD (58.36%, 58.63%, and 58.06%, respectively), whereas the lowest prevalence was observed for C1 (48.07%). The prevalence of MAFLD was lower in women than in men (20.88% vs. 54.37%, *p* < 0.001). In women, a very similar prevalence of MAFLD was found in C_F_1, C_F_3, and C_F_4, whereas C_F_2 had the highest prevalence of MAFLD (26.92%).

The results of the binary logistic regression models are presented in Table 3, stratified by sex. C1 and C_F_1 were chosen as the reference clusters for males and females because they had the largest populations and showed a lower prevalence of MAFLD compared with the other clusters. After adjusting for confounding variables, C2, C6, C8, and C9 among males were significantly associated with a higher risk of MAFLD. C3, C4, and C7 had significantly lower odds of belonging to the MAFLD group. In the female clusters, the odds of C_F_2 belonging to MAFLD were higher (AOR = 1.370, 95% CI = 1.168–1.607).

**Table 3:** Associations between MAFLD and lifestyle clusters

Male-Clusters	Unadjusted Model	*P*	Adjusted Model	*P*
	OR (95% CI)		AOR (95% CI)	
Cluster 1	Ref		Ref	
Cluster 2	1.076(1.028–1.127)	0.002	1.191(1.136–1.248)	< 0.001
Cluster 3	1.260(1.201–1.321)	< 0.001	1.243(1.184–1.304)	< 0.001
Cluster 4	1.514(1.453–1.578)	< 0.001	1.455(1.395–1.516)	< 0.001
Cluster 5	1.125(1.051–1.204)	< 0.001	0.987(0.919–1.059)	0.710
Cluster 6	1.277(1.212–1.345)	< 0.001	1.375(1.304–1.450)	< 0.001
Cluster 7	1.531(1.471–1.594)	< 0.001	1.508(1.448–1.571)	< 0.001
Cluster 8	1.184(1.077–1.301)	< 0.001	1.209(1.097–1.333)	< 0.001
Cluster 9	1.495(1.437–1.556)	< 0.001	1.611(1.547–1.678)	< 0.001
Female-Clusters	Unadjusted Model	*P*	Adjusted Model	*P*
	OR (95% CI)		AOR (95% CI)	
Cluster1	Ref		Ref	
Cluster 2	1.403(1.222–1.610)	< 0.001	1.452(1.252–1.683)	< 0.001
Cluster 3	1.014(0.846–1.214)	0.883	1.296(1.070–1.570)	0.008
Cluster 4	0.997(0.936–1.063)	0.937	1.060(0.991–1.133)	0.091

Adjusted Model, adjusted for age, education level, marital status, occupational category, and type of medical insurance

## Discussion

Using the analysis of the largest datasets of Chinese adults aged 18–79 years with physical examinations to date, we established 13 distinct lifestyle patterns and linked the patterns with MAFLD. Our results suggested that the lifestyle patterns from real-world data could be new indices to understand and track the cumulative effect of healthy and unhealthy lifestyle behaviours on overall health risk in MAFLD populations, although the HCSAQ item-based lifestyle scoring system in this study has some limitations compared with existing well-established health-promoting lifestyle profiles [26, 27].

To our knowledge, the current study is the first to propose lifestyle patterns that include mental health behaviours and a broad range of lifestyle variables. While the clustering of smoking, excessive alcohol consumption, diet, physical activity, sedentary lifestyle, and sleep behaviours has been common in previous studies of lifestyle patterns [28–30], few studies have included mental health or all lifestyle behaviours. Given that mental health is also an important component of lifestyle [15], we integrated mental health behaviours into our analysis and derived lifestyle patterns together with other lifestyle behaviours, which is important to establish a clear picture of their interplay and associations with MAFLD. Furthermore, we conducted a sex-stratified analysis of lifestyle patterns, which could help to develop targeted interventions for different groups of behavioural clusters.

Almost half of the individuals in the present study had lifestyle patterns with varying degrees of coexisting healthy and unhealthy behaviours, such as no smoking or drinking, physical inactivity, a highly sedentary lifestyle, a poor mentality, and an unhealthy diet in C2 (men). This finding is consistent with previous studies and suggests that these individuals are potential targets for future health education and behavioural interventions [8]. Among previously published studies, lifestyle interventions for MAFLD patients have been focused on diet and/or exercise [31, 32], but the combined effect of all lifestyle behavioural interventions in MAFLD remains unclear. Considering these results, we recommend focusing on as many lifestyle behaviours as possible in combination, rather than just one or a few common health-related behaviours, to improve public health to a greater extent.

Compared with C1, the prevalence of MAFLD was higher in the remaining eight lifestyle patterns of men, and C_F_2 was more prevalently associated with MAFLD than C_F_1 in the female groups. This finding may suggest that the synergistic effect of having high levels of physical activity and high-quality sleep, combined with a healthy mentality and diet and in the absence of smoking and alcohol consumption, is associated with a lower risk of MAFLD. The potential cumulative effects of these behaviours on adults can be explained. Although exercise and diet are important public health priorities, sleep and mental health are often overlooked. Growing evidence shows that poor sleep and mental health are associated with MAFLD [33, 34], which this study seems to confirm. Given their potential impact on MAFLD, future studies should comprehensively assess sleep and mental features to explore their relationship with MAFLD, and greater efforts should be made to raise awareness of strategies to improve sleep quality and mental health. Our findings also showed that most lifestyle patterns associated with an increased risk of MAFLD in men were characterized by smoking and/or alcohol consumption, whether combined with other healthy/unhealthy behaviours. Therefore, heavy smoking and/or heavy alcohol consumption may obscure the potential positive effects of a healthy lifestyle on reducing MAFLD risk [16]. This “offsetting” effect of one specific behaviour on another has been previously reported in studies [35]. Our results suggest that maintaining good lifestyle habits does not alleviate the effect of smoking and excessive alcohol consumption on MAFLD.

The sex differences in lifestyle patterns are reflected in two aspects. On the one hand, the number of derived clusters is unequal, suggesting that women’s lifestyles are more homogeneous than men who have multiple combinations of lifestyle behaviours to varying degrees. On the other hand, the percentage of people with healthy lifestyle patterns is different; less than one in five men (19.91%) have a healthy lifestyle pattern, whereas most women (90.93%) have a healthy lifestyle pattern, which indicates that unfavourable clusters of lifestyle behaviours are more prominent in men. Therefore, future lifestyle interventions would need to focus more on men. Furthermore, certain behaviours in lifestyle patterns also have significant sex differences. For example, clusters containing alcohol and tobacco consumption are more prevalent in men than in women, which may be related to the fact that, in China, men are often under high stress and prone to adopt unhealthy lifestyles, such as irregular work, social drinking, and smoking [36]. However, men are more motivated to stay physically active than women, which is consistent with global trends [37]. Other aggregated behaviours, including various dietary behaviours, were similar between men and women, but adherence to healthy dietary behaviours was generally higher among women than men, which is consistent with previous research [38]. Some suggestions about public health should be submitted to the government so that policies and behaviour change strategies will encourage women to be more physically active and help men spend more time on dietary improvements and stress relief.

Our results showed that the prevalence of MAFLD varies widely between males and females. The prevalence of MAFLD in males was 54.37%, which was significantly higher than that in females (20.88%), which was similar in other regions of China [3, 39]. Most importantly, the risk of MAFLD between lifestyle patterns was significantly different in men and women. Among men, all lifestyle patterns were associated with a higher risk of MAFLD, whereas the risk of MAFLD observed in the female lifestyle patterns was much lower than the overall risk. These accurate MAFLD risk assessment results were not found in previous studies, which we speculate to be related to lifestyle choices influenced by social, cultural, and economic contexts and to metabolic disorders related to genetic and hormonal regulation. As Karthickeyan Krishnan said, inequalities in health between men and women can be explained by sex-related biological differences, genetic predispositions, and lifestyle factors [40]. Further studies are needed to elucidate the mechanisms of these sex differences. In addition, future research should attempt to focus on population information from various perspectives, such as liver disease-related outcomes, age and menopausal status, to assess the degree of MAFLD risk as accurately as possible [41] and identify different intervention methods for differentiated clusters accordingly.

### Limitations

Our study has several limitations. First, lifestyle information was self-reported data that may lead to recall bias. However, lifestyle items were derived from the HCSAQ, which is widely used in Chinese health examination facilities because of its validity and reliability. Moreover, the cross-sectional design used in this study hinders the inference of causal relationships between lifestyle patterns and MAFLD, and further prospective studies should be conducted. But the applied design was effective in the identification of lifestyle patterns. Secondly, MAFLD was diagnosed using ultrasound methods rather than histological assessments. Nevertheless, ultrasound methods are widely used for population-based studies. Thirdly, we lacked more detailed indices for assessing liver function or liver fibrosis index since this study was based on retrospective health physical examination data, which resulted in the possibility of MAFLD not being accurately assessed. Finally, we were unable to exactly assess the independent effects of alcohol consumption on MAFLD, and we explored interactions between dietary and mental health variables through principal component analysis, but did not have detailed information regarding the analysis of interactions between other lifestyle variables, which we will focus on in our next study.

## Conclusions

In conclusion, the lifestyle patterns derived from our study identify differences in lifestyle behaviours between Chinese men and women and contribute to a broader understanding of the interrelationships between lifestyle patterns and MAFLD. Men are less likely to adhere to healthy lifestyles, and their lifestyle patterns place them at higher risk of developing MAFLD than women, highlighting the need for person-centred public health policies to address health inequities. Furthermore, developing evidence-based lifestyle intervention strategies based on culture, norms, and values for specific population subgroups and facilitating their implementation in the health care system are needed to prevent the increased prevalence of MAFLD.

### Supplementary Information

**Additional file 1: Table S1.** The lifestyle scoring system. **Table S2.** Factor load of dietary factors*.

## Data Availability

The datasets used and/or analysed during the current study are available from the corresponding author upon reasonable request.
