# Supplementary material for: Clustering of lifestyle behaviours and analysis of their associations with MAFLD: a cross-sectional study of 196,515 individuals in China
Source: BMC Public Health. 2023 Nov 21;23:2303. doi: 10.1186/s12889-023-17177-3 (PMC10664514; doi:10.1186/s12889-023-17177-3)
Supplement: Supplementary file 1 — Additional file 1: Table S1. The lifestyle scoring system. Table S2. Factor load of dietary factors*. [file 12889_2023_17177_MOESM1_ESM.doc]

**Table S1.** The lifestyle scoring system

| Lifestyle items (Mainly surveyed in the last month) | Options | Assignment |
| --- | --- | --- |
| 1. Do you usually eat three meals on time? | Unable (>3 days/week unable to take meals on time) | 0 |
|  | Basically able (unable to eat on time 2~3 days/week) | 1 |
|  | Able (able to eat on time almost every day) | 2 |
| 2. Do you often eat late-night snacks? | No | 2 |
|  | Occasionally (≤1 time/week) | 1 |
|  | Frequently (>1 time/week) | 0 |
| 3. Do you often overeat? | Yes | 0 |
|  | No | 1 |
| 4. How often do you participate in social gathering? | No or occasional participation (≤ 1 time/week) | 2 |
|  | More often (2~3 times/week) | 1 |
|  | Frequently (4~5 times/week) | 0 |
|  | Very frequent (>5 times/week) | 0 |
| 5. What is your dietary taste? | Light | 2 |
|  | Salty | 0 |
|  | Not sure | 1 |
| 6. Do you have dietary preferences (for example, preference for desserts, fast food, etc.)? | Yes | 0 |
|  | No | 1 |
| 7. What is the structure of your staple diet? | Mainly refined grain | 0 |
|  | Mixing of coarse and refined grains | 1 |
|  | Mainly coarse grains | 1 |
| 8. How often do you drink milk? | No | 0 |
|  | Occasionally (1~2 times/week) | 1 |
|  | Frequently (3~5 times/week) | 2 |
|  | Every day (>5 times/week) | 2 |
| 9. How often do you eat eggs? | No | 0 |
|  | Occasionally (1~2 times/week) | 0 |
|  | Frequently (3~5 times/week) | 1 |
|  | Every day (>5 times/week) | 2 |
| 10. How often do you eat beans and soy products? | No | 0 |
|  | Occasionally (1~2 times/week) | 1 |
|  | Frequently (3~5 times/week) | 2 |
|  | Every day (>5 times/week) | 2 |
| 11. How often do you eat fruits? | No | 0 |
|  | Occasionally (1~2 times/week) | 1 |
|  | Frequently (3~5 times/week) | 2 |
|  | Every day (>5 times/week) | 2 |
| 12. How much vegetables do you eat on average per day? | <100g | 0 |
|  | 100~200g | 1 |
|  | 200~500g | 2 |
|  | >500g | 2 |
| 13. How much red meat (pork, beef, lamb, poultry) do you eat on average per day? | <50g | 1 |
|  | 50~100g | 2 |
|  | 101~250g | 0 |
|  | >250g | 0 |
| 14. How often do you eat fatty meat? | No | 1 |
|  | Occasionally (1~2 times/week) | 2 |
|  | Frequently (3~5 times/week) | 0 |
|  | Every day (>5 times/week) | 0 |
| 15. How often do eat animal offal? | No | 1 |
|  | Occasionally (1~2 times/week) | 2 |
|  | Frequently (3~5 times/week) | 0 |
|  | Every day (>5 times/week) | 0 |
| 16. How often do you eat fish or seafood? | No | 0 |
|  | Occasionally (1~2 times/week) | 1 |
|  | Frequently (3~5 times/week) | 2 |
|  | Every day (>5 times/week) | 2 |
| 17. How often do you drink coffee? | No | 0 |
|  | Occasionally (1~2 times/week) | 1 |
|  | Frequently (3~5 times/week) | 2 |
|  | Every day (>5 times/week) | 2 |
| 18. How often do you drink sugary drinks (colas, etc.)? | No | 2 |
|  | Occasionally (1~2 times/week) | 1 |
|  | Frequently (3~5 times/week) | 0 |
|  | Every day (>5 times/week) | 0 |
| 19. Do you smoke? (Refers to continuous smoking for more than 1 year) | No | 1 |
|  | Smoking every day | 0 |
|  | Quit smoking (have quit smoking for a year or more) | 1 |
|  | Passive smoking (cumulative 15 minutes or more per day and more than 1 day per week) | 0 |
| 19-1. How many cigarettes do you usually smoke per day? (For those who have quit smoking, please answer the number of cigarettes smoked per day before quitting) | <10 cigarettes | -1 |
|  | 10~20 cigarettes | -2 |
|  | 21~30 cigarettes | -3 |
|  | >30 cigarettes | -3 |
| 19-2. How many years have you been smoking? (For those who have quit smoking, please answer the number of years of smoking before quitting) | <5 years | -1 |
|  | 5~10 years | -1 |
|  | 11~20 years | -2 |
|  | >20 years | -3 |
| 19-3. How long have you quit smoking? | <1 year | -2 |
|  | 1~5 years | -2 |
|  | 6~10 years | -2 |
|  | >10 years | -1 |
| 20. Do you drink alcohol? (Refers to drinking more than 150ml of white wine per week on average) | No | 2 |
|  | Yes | 0 |
|  | Used to drink, now quitting (have quit drinking for a year or more) | 1 |
| 20-1. How many times a week do you drink alcohol? (For those who have quit drinking, please answer the number of times you drank per week before quitting) | 1~2 times | -1 |
|  | 3~5 times | -2 |
|  | >5 times | -3 |
| 20-2. How much white wine do you drink each time? | <100 ml | -1 |
|  | 100~200 ml | -2 |
|  | >200 ml | -3 |
| 20-3. How many years have you been drinking? (For those who have quit drinking, please answer the number of years of drinking before quitting) | <5 years | -1 |
|  | 5~10 years | -2 |
|  | 11~20 years | -2 |
|  | >20 years | -3 |
| 20-4. How long did you quit drinking? | <1 year | -3 |
|  | 1~5 years | -3 |
|  | 6~10 years | -2 |
|  | >10 years | -1 |
| 21. Do you participate in physical exercise? (Refers to exercising once or more per week) | No | 0 |
|  | Yes | 1 |
| 21-1. How many times per week do you exercise? | 1~2 times | 1 |
|  | 3~5 times | 2 |
|  | >5 times | 3 |
| 21-2. How much time do you spend on each exercise routine? | <30 minutes | 1 |
|  | 30~60 minutes | 2 |
|  | >60 minutes | 3 |
| 21-3. How many years have you exercised? | <1 year | 1 |
|  | 1~5 years | 1 |
|  | 6~10 years | 2 |
|  | >10 years | 2 |
| 22. How physically intense is your work? | Not working | 0 |
|  | Mainly brain work | 2 |
|  | Light labour | 2 |
|  | Moderate physical work | 1 |
|  | Heavy labour | 1 |
| 22-1. How many days do you work per week? | <3 days | 0 |
|  | 3~5 days | 2 |
|  | >5 days | 1 |
| 22-2. How long do you work on average per day? | <4 h | 1 |
|  | 4~6 h | 2 |
|  | 6~8h | 2 |
|  | >8h | 0 |
| 23. Apart from work and study time, how much time do you spend sitting every day (such as watching TV, surfing the Internet)? | <2h | 2 |
|  | 2~4h | 2 |
|  | 4~6h | 1 |
|  | >6h | 0 |
| 24. How was your sleep quality in the last month? | Good | 2 |
|  | Common | 1 |
|  | Poor | 0 |
| 25. What is the average time you sleep per day? | <5h | 0 |
|  | 5~7h | 1 |
|  | 7~9h | 2 |
|  | >9h | 2 |
| 26. Do you feel sullen and depressed? | None | 2 |
|  | Occasionally | 1 |
|  | Often | 0 |
| 27. Do you get emotional or angry easily? | None | 2 |
|  | Occasionally | 1 |
|  | Often | 0 |
| 28. Do you feel nervous and find it hard to relax? | None | 2 |
|  | Occasionally | 1 |
|  | Often | 0 |
| 29. Do you tend to be more nervous and anxious than usual? | None | 2 |
|  | Occasionally | 1 |
|  | Often | 0 |
| 30. Do you lose your temper easily and have no patience? | None | 2 |
|  | Occasionally | 1 |
|  | Often | 0 |
| 31. Do you feel exhausted and unenthusiastic about people and things? | None | 2 |
|  | Occasionally | 1 |
|  | Often | 0 |
| 32. Do you get anxious and distracted easily? | None | 2 |
|  | Occasionally | 1 |
|  | Often | 0 |
| 33. Do you feel depressed or frustrated? | None | 2 |
|  | Occasionally | 1 |
|  | Often | 0 |
| 34. Do you have difficulty concentrating? | None | 2 |
|  | Occasionally | 1 |
|  | Often | 0 |

**Table S**2. Factor load of dietary factors*

| Dietary Factor 1 | | Dietary Factor 2 | | Dietary Factor 3 | | Dietary Factor 4 | | Dietary Factor 5 | |  |
| --- | --- | --- | --- | --- | --- | --- | --- | --- | --- | --- |
| Food | Factor | Food | Factor | Food | Factor | Food | Factor | Food | Factor |  |
| Milk | 0.47 | Dietary tastes | 0.75 | Three meals on time | 0.42 | Overeat and overdrink | 0.60 | Fatty meat | 0.76 |  |
|  |
| Eggs | 0.62 | Dietary preference | 0.73 | Late-night snacks | 0.52 | Social intercourse | 0.68 | Animal offal | 0.74 |  |
|  |
| Beans and soy products | 0.63 | Staple food structure | 0.45 | Coffee | -0.62 | Red Meat | 0.41 |  |  |  |
|  |
| Fruits | 0.54 |  |  | Sugary drinks | 0.70 |  |  |  |  |  |
|  |
| Vegetables | 0.42 |  |  |  |  |  |  |  |  |  |
| Fish/seafood | 0.54 |  |  |  |  |  |  |  |  |  |

**Note:**

*Only factor loadings >0.4 are shown in the table

*Eighteen culture-specific items regarding dietary intake and dietary habits were entered into a principal exploratory factor (PCA) analysis. Orthogonal rotation (varimax option) was used to obtain uncorrelated patterns with greater interpretability. The Kaiser-Meyer-Olkin (KMO) test statistic of 0.762 and Bartlett's spherical test p<0.001 supported the appropriateness of factor analysis. There were five factors with eigenvalues >1, which contributed 44.39% to the total variance. Similarly, PCA was performed on nine mental health items. The KMO test statistic was 0.934 and Bartlett's spherical test p < 0.001, suitable for factor analysis. There was one factor with an eigenvalue > 1, and its contribution to the total variance was 63.52%.
